# To the Editors of the Medical and Physical Journal

**Published:** 1809-10

**Authors:** J. Edwards

**Affiliations:** London


### *To the Editors of the Medical and Physical Journal.*

GENTLEMEN,

BEING well convinced of the great circulation of your publication, the Medical and Physical Journal, I request your insertion of the following, as it probably may corroborate the opinions of several, and in some measure dissipate the prejudice of others. Having attentively listened to the various opinions of many professional men, as to the effects produced by "Venereal Virus," I resolved to try experiments on myself, judging that that would be the most certain means of satisfying a dubious mind; I therefore took from the chancre of a patient as much virus as I could procure, and applied it to my urethra. In the course of twenty-four hours I was attacked with the usual symptoms of a confirmed gonorrhœa. Being perfectly satisfied that the virus of a chancre would produce gonorrhœa, I determined, (after being cured of the effects of my former experiment) vice versa, to try whether the virus of gonorrhœa would produce chancre; I therefore obtained some gonorrhœal virus, and applied it behind the preputium. I found, in the course of a few days, small excrescences, afterwards an ulcer, though not much resembling that of a chancre. I did not interfere with it; and as I perceived that it neither increased nor occasioned any bubo, it led me to conclude, that the virus only acted as a corroding matter, not near so virulent in its effects as that of a confirmed Syphilis.

I am, &c.

*London, Sept.* 8, 1809. J. EDWARDS.

---

The Reader will find experiments, with a somewhat similar result, in an Inaugural Dissertation published by Dr. W. Harrison, and inserted in the London Medical Journal. The only difference is, that in Dr. H's experiments, gonorrhœal matter induced no irritation. In Mr. Hunter's experiments, the gonorrhœal matter induced chancre.—Mr. Howard is at a loss to reconcile Mr. Hunter's experiment with Dr. Harrison's; Mr. Edwards's might seem to increase the difficulty: but it is more to the purpose to mark the agreement of all three in the effect of chancrous matter applied to the urethra. That the application of matter from three different gonorrhœas should produce three different effects, proves only what has been often remarked, remarked, and what must occur to every thinking mind, viz. that there are gonorrhœas from many causes besides the venereal poison, and that some of these are contagious, others not; but that only the venereal gonorrhœa will induce chancre. This subject is considered very much at large in the new edition of Mr. Hunter's work. A.

